# Study of the Sensitivity Limit of Detection of α-Particles by Polymer Film Detectors LR-115 Type 2 Using X-ray Diffraction and UV-Vis Spectroscopic Methods

**DOI:** 10.3390/polym15112500

**Published:** 2023-05-29

**Authors:** Dana S. Yerimbetova, Artem L. Kozlovskiy, Umitali N. Tuichiyev, Kassym S. Zhumadilov

**Affiliations:** 1 Engineering Profile Laboratory, L. N. Gumilyov Eurasian National University, Astana 010008, Kazakhstan; 2 Laboratory of Solid State Physics, Institute of Nuclear Physics, Almaty 050032, Kazakhstan; 3 Department of “Chemical Processes and Industrial Ecology”, Satbayev University, Almaty 050032, Kazakhstan; 4 RSE “Medical Centre Hospital of the President’s Affairs Administration of the Republic of Kazakhstan”, Astana 010008, Kazakhstan

**Keywords:** solid-state nuclear track detectors LR-115 type 2, α-particles, optical spectroscopy, X-ray diffraction, radon decay products, dosimetry

## Abstract

This work is devoted to the applicability assessment of optical spectroscopy and X-ray diffraction methods to establish the lower detection limit for the density of latent tracks from α-particles in polymer nuclear-track detectors, in the case of simulation of the formation of radon decay daughter products using Am-241 sources. During the studies, the detection limit for the density of latent tracks—traces of the interaction of α-particles with the molecular structure of film detectors—was established using optical UV spectroscopy (10^4^ track/cm^2^) and X-ray diffraction (10^4^ track/cm^2^). At the same time, analysis of the connection between structural and optical changes in polymer films indicates that a growth in the density of latent tracks above 10^6^–10^7^ results into the formation of an anisotropic change in the electron density associated with distortions in the molecular structure of the polymer. An analysis of the parameters of diffraction reflections (the position and width of the diffraction maximum) showed that in the range of latent track densities of 10^4^–10^8^ track/cm^2^, the main changes in these values are associated with deformation distortions and stresses caused by ionization processes during the interaction of incident particles with the molecular structure of the polymer. The increase in optical density, in turn, is caused by the accumulation of structurally changed regions (latent tracks) in the polymer as the irradiation density increases. A general analysis of the obtained data showed good agreement between the optical and structural characteristics of the films depending on the irradiation density.

## 1. Introduction

As is known, the sources of natural radioactivity in most cases are cosmic rays penetrating the Earth through the atmosphere in the form of streams of charged particles, gamma quanta, or electromagnetic radiation, as well as various radionuclides present in the environment, water, soil, and rocks [1,2,3]. At the same time, the distribution of the radiation background on the Earth is not isotropic in nature, there are areas with an increased background radiation and radiation levels, and in most cases, this is not associated with any man-made disasters or human activities, but are of natural origin [4,5]. As a rule, the formation of such areas depends on several factors, including the location above sea level, the composition of the soil, or the presence of deposits of uranium or other radioactive elements [6,7].

Natural radionuclides can be classified as primary radionuclides that have been present on the Earth for quite a long time (^40^K, ^87^Rb), which are decay products of uranium (^238^U) or thorium (^232^Th). It is worthy to note that all isotopes formed during natural radioactive decays are solids, except for radon (Rn), which is a noble radioactive gas. At the same time, radon occurs in nature in the form of several isotopes: radon—222, radon—220, and radon—219, formed subsequent to radioactive decays of uranium and thorium. Radon itself is formed during the natural radioactive decay of radium, which is formed subsequent to the radioactive decay of uranium [8,9,10]. At the same time, the ubiquity of radon is primarily due to the fact that small amounts of uranium are found almost everywhere in the thickness of the earth’s crust, as well as in soil or stone deposits. To date, the International Agency for Research on Cancer, the World Health Organization, and the International Atomic Energy Agency classify radon as one of the most dangerous elements for human health [11,12,13].

One of the most common methods for determining the concentration of radon and its daughter decay products in the form of α-particles, due to the fact that radon itself is a radioactive noble gas, is the method of using solid-state nuclear track detectors. The basic principle of using these detectors is to detect radon decay products in the form of α-particles with an energy of more than 5 MeV and a sufficiently high penetrating power in air (up to several centimeters) [14,15]. At the same time, due to the fact that radon is colorless and odorless, it is impossible to detect its presence in rooms or in places of its formation [16]. The main advantage of the method for determining the concentration of radon and its daughter decay products is the possibility of simultaneous exposure of several detector samples and their centralized chemical processing, as well as the ability to take into account daily and seasonal changes in concentrations, which is impossible with “instantaneous” measurements. All this makes it possible to avoid the large-scale financial and time costs required to determine the dynamics of changes in the radiation background. Additionally, a distinctive feature of radon registration is the fact that it is the only gas whose decay product is α-particle with high energy [17,18].

At the same time, in the case of measuring daughter decay products in the form of α-particles, one should consider not only the maximum possible path length of particles in air, but also the penetrating ability of α-particles in the detector material. In most cases, it does not exceed 20–70 microns, depending on the initial energy of α-particles. The very method of measuring volumetric activity using film detectors includes several stages: sampling or long-term exposure of detectors in the measured room, film etching to visualize latent tracks and subsequent determination of their density, as well as the use of recalculation formulas to determine the concentration of radon and its daughter decay products. At the same time, the duration of exposure directly affects the accuracy of determining volumetric activity, as well as the establishment of concentration dependences.

To date, a large number of studies are known on the use of various types of polymer films or solid-state nuclear track detectors for changing background radiation, fission fragments, various types of exposure, etc. The most complete information about the applicability of various types of solid-state nuclear track detectors as various practical applications is given in [19,20]. According to these works, in recent years, much attention has been paid to the use of solid-state nuclear track detectors to determine the daughter products of radon decay, as well as to determine its concentration in order to monitor the environmental situation in various regions. However, the use of film detectors in the case of high radon concentration or long exposure time is not always effective, since, when visualizing latent tracks, chemical etching of polymer films can lead to the formation of the effect of overlapping latent tracks and the impossibility of a direct accurate calculation of their density. In this regard, there is a problem of accurate determination of volumetric activity, as well as a large measurement error, which is unacceptable in regions with an increased radiation background. To solve this problem, much attention is paid to the search for new ways to determine the density of latent tracks without chemical etching of latent tracks, as well as their visualization. Determination of the density of latent tracks, as well as changes in the properties of polymer films, is a very important condition in determining the potentials for using polymers not only as detecting systems, but also as various sensors, etc. [21,22].

The relevance of this study consists in the development of alternative methods for non-destructive solid-state nuclear track detectors due to chemical etching, determination of volumetric activity based on changes in structural parameters and optical characteristics, as well as in the possibility of determining the accumulated dose of radiation in the case of a long exposure, in which direct visual counting of tracks is impossible, due to the effect of overlapping of etched latent tracks, which makes it difficult to accurately determine their density. The need to study the applicability of optical spectroscopy and X-ray diffraction methods for estimating the lower limit of detection of the density of latent tracks is due to several factors. Firstly, most works devoted to the study of latent tracks using optical spectroscopy or X-ray diffraction methods [23,24,25,26] consider the effects of deep overlap of latent tracks (radiation density above 10^10^ track/cm^2^), in cases where there are quite a lot of structural changes in the polymer film, which results into serious changes in properties. Secondly, in the case of using polymer films as detectors to determine the concentration of radon or its daughter decay products (α-particle), there is a need for calibration curves, the use of which will allow the films not to be etched and reused to determine the temporal concentration dependences. Thirdly, the use of these methods allows obtaining new dependences on the connection between structural changes in the molecular structure of polymer films depending on the irradiation density, as well as their optical properties and optical density.

## 2. Experimental Part

Standard solid-state nuclear track detectors LR-115 type 2 manufactured by Dosirad (Paris, France) were used as objects of study to determine the daughter products of radon decay, as well as to determine the dose dependence when the detectors were exposed to α-sources. The LR-115 type 2 film detector is a polymer film based on cellulose nitrate (C_6_H_7_(NO_2_)_3_O_5_)*_n_*, soluble in acetone, and also not resistant to alkalis, which makes it possible to etch latent tracks formed in it upon interaction with deposited charged particles. The density of the films is 1.58–1.65 g/cm^3^, and the melting point is 160–170 °C.

Americium-241 (Am-241) was chosen as a source for simulation of alpha radiation with an energy characteristic of the radon decay daughter products. The main channel for the production of α-particles can be written as follows: ^241^Am → ^237^Np + α (5.6 MeV). The nominal activity of the source Am-241 was 338 Bq (T_1/2_ = 432.6 years), produced by STC “RADEK” (serial number No. 0241-06/17). The exposure time of the samples was chosen experimentally considering the need to obtain different densities of latent tracks in polymer detectors to determine the dose dependence of changes in structural and optical characteristics, as well as to establish the limits of determination and applicability of X-ray diffraction and optical spectroscopy methods for determining the absorbed dose and the density of latent tracks. The total duration of the experiments was more than 1 year, which made it possible to obtain a number of different dose dependences for comparative analysis.

UV spectroscopy and X-ray diffraction were used as the main research methods for assessment of changes in solid-state nuclear track detectors exposed to the daughter products of radon decay.

To determine the influence of the accumulation of the absorbed dose and caused by the interaction of α-particles with the polymer structure of the nuclear track detector on the optical properties, a technique was used to assess changes in the transmission of optical spectra in a wide range of wavelengths. Based on the obtained data of the optical transmission spectra, the optical characteristics of the studied samples were determined depending on the accumulated radiation dose.

The X-ray diffraction method and X-ray diffraction analysis were used to determine the structural distortions caused by track formation in polymer films during the interaction of α-particles with the polymer structure and the consequences in the form of changes in the molecular structure of the polymer. For analysis, the classical Bragg–Brentano geometry for X-ray diffraction patterns in the angular range of 2θ = 10–60°, with a step of 0.03° was used.

To visualize latent tracks from α-particles, a standard chemical etching method was used, followed by an assessment of the track density using scanning electron microscopy.

## 3. Results and Discussion

One of the key conditions for the applicability of various detection methods for determining dose loads or absorbed dose is the sensitivity limit of methods that allow determining the minimum detectable doses. In the case of traditional methods for determining the absorbed dose using the chemical etching technique and subsequent direct counting of visually developed latent tracks in polymer detectors, the sensitivity limit is highly dependent on the resolution of microscopy, as well as the geometry of the etched latent tracks. As a rule, to determine the density of latent tracks, scanning electron microscopes are used with a resolution of up to 10,000×–20,000× magnification, which makes it possible to estimate the density of latent tracks with high accuracy even with small radii (less than 100 nm) [27,28,29].

**Figure 1:**
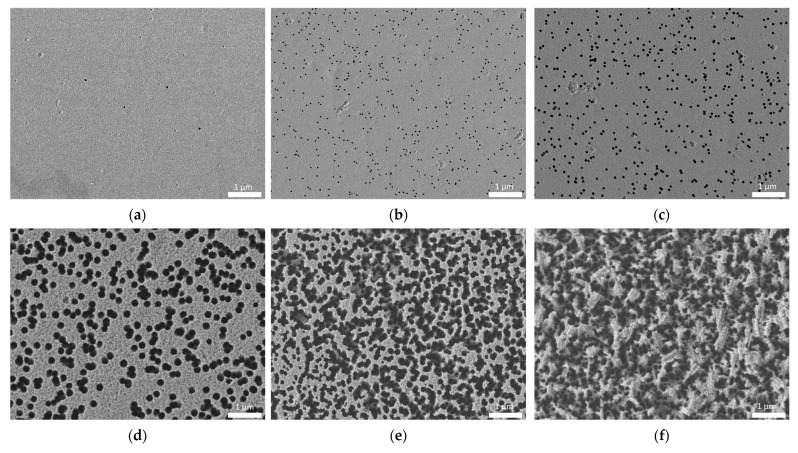
Examples of images of the surface of polymer films irradiated with charged particles with different densities: (**a**) less than 10^2^ pores/cm^2^; (**b**) 10^4^ pores/cm^2^; (**c**) 10^6^ pores/cm^2^; (**d**) 10^8^ pores/cm^2^; (**e**) 10^9^ pores/cm^2^; (**f**) 10^10^ pores/cm^2.^

**Figure 2:**
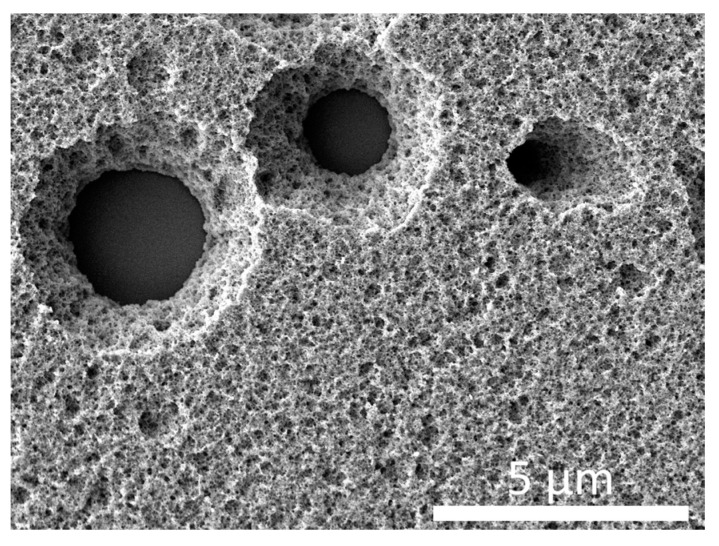
An example of an etched film to reflect the latent track geometry at low densities.

In this case, in addition to the minimum density of latent tracks visualized by chemical etching, there are limitations within the limits of the maximum values of the absorbed dose and, accordingly, the track density. These limitations are primarily related to the formation of overlapping latent tracks after their chemical etching for imaging. As a rule, a similar limitation for polymer films subjected to irradiation is imposed for latent track densities above 5 × 10^7^–1 × 10^8^ track/cm^2^, in view of the fact that for direct counting using high-resolution scanning electron microscopes, it is necessary that the track diameter be more than 200–300 nm. At densities above 5 × 10^7^ track/cm^2^, this can lead to regions with crossing diameters or overlapping track diameters, which in turn makes direct machine counting difficult and requires a clear definition of the number of overlap areas. In the case of densities above 1 × 10^8^ track/cm^2^, for a detailed assessment of the density of latent tracks in order to avoid the formation of overlapping regions, the track diameters should not exceed 100–150 nm. At the same time, even in this case, the probability of the presence of overlapping regions remains due to the chaotic effect of the interaction of α-particles with the polymer structure, which at high densities may consist in the probability of two α-particles getting close enough to each other. This, in turn, will lead to the formation of overlapping track diameters or their merging into one larger track during chemical etching. For densities above 1 × 10^10^ track/cm^2^, the visualization of latent tracks and direct calculation of their density using scanning electron microscopy is very difficult due to the large number of areas of overlap during etching, even in the case of diameters less than 100 nm, the formation of which is associated with a growth in the probability of two charged particles hitting one point at high densities. Figure 1 shows examples of determining the densities of latent tracks in polymer films at different densities, as well as those reflecting the formation of latent track overlap effects in the case of high track densities (10^9^–10^10^ tracks/cm^2^). As can be seen from the data presented, at low densities of latent tracks, the number of pores in the area under study is rather small, which makes it difficult to accurately determine the density and results into the need to etch latent tracks to large diameters, which makes it possible to obtain images at low magnifications covering a large area of the sample. An example of such etching is shown in Figure 2, according to which it can be seen that the detected α-particle completely passes through the entire thickness of the detector (12 µm), which in turn indicates a high penetrating ability of α-particles in the polymer.

As can be seen in the presented images of Figure 1d, a growth in density to 10^8^ pores/cm^2^ results into the appearance of the effect of the formation of crossed pores located close to each other and, due to large diameters, they overlap. As is evident from the data in Figure 1e,f, with a growth in density above 10^9^ pores/cm^2^, this does not allow separation of individual pores due to the large overlap.

Thus, by analyzing the obtained data of images of the surface of polymer films exposed to α-particles with different densities, we can conclude that the use of scanning electron microscopy methods for direct calculation of latent tracks and their density has its limitations at densities above 10^8^ pores/cm^2^, and in the case of densities above 10^10^, direct counting of pores is not possible using classical methods for determining pore density.

**Figure 3:**
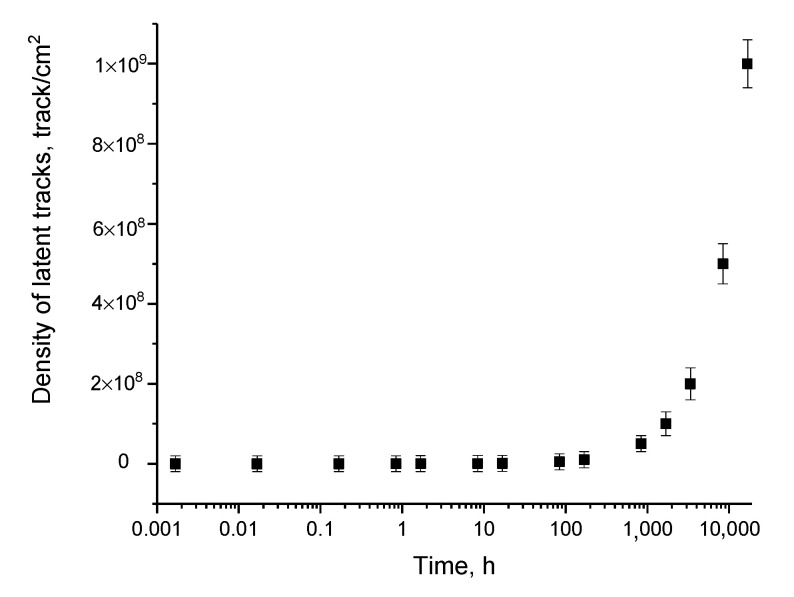
Dependence of the change in the density of latent tracks on the exposure time of film detectors when they are placed on the Am-241 source.

Based on the images obtained using the scanning electron microscopy of film detectors after specified exposure times during irradiation of samples with an Am-241 source, which makes it possible to model the daughter products of radon decay in the form of α-particles with an energy of more than 5 MeV, the dependence of the pore density on the exposure time was plotted. Results of the construction are presented in Figure 3. As is evident from the dependence acquired, with a growth in the density of recorded latent tracks, the measurement error and the rms standard deviation in the calculation increase, which is associated with the effect of overlapping latent tracks during their etching [24,25,26]. In [24,25,26], the effect of the appearance of overlapping latent tracks was considered in detail, according to the calculated estimates of which, in the unetched state, the sizes of the changed regions range from 5 to 20 nm, and the regions themselves are at a fairly close distance from each other, which in the case of chemical etching will lead to their overlap. It should also be noted that for high densities, in addition to a growth in the measurement error due to the formation of areas of overlap of etched latent tracks, the destruction of the polymer itself after etching is observed due to the etching of most of the damaged polymer in the form of latent tracks, which somewhat complicates further work with these films (see the data in Figure 1e,f).

To determine the density of latent tracks formed during the interaction of incident particles with the molecular structure of polymer films, in a number of works [23,24] it is proposed to use optical spectroscopy methods, in particular, by analyzing changes in optical spectra caused by irradiation. Thus, the authors of [23] showed that the shift of the fundamental absorption edge of polymer films subjected to irradiation characterizes the change in the electron density and its anisotropy in the region of the formation of latent tracks, which the authors attribute to a change not only in the band gap, but also in the formation of a depleted region along trajectories of motion of charged particles in the material. Thus, the authors of [25] revealed that the shift of the fundamental absorption edge of polymer films subjected to irradiation characterizes the change in the electron density and its anisotropy in the region of the formation of latent tracks, which the authors attribute to a change not only in the band gap, but also in the formation of a depleted region along trajectories of motion of charged particles in the material.

**Figure 4:**
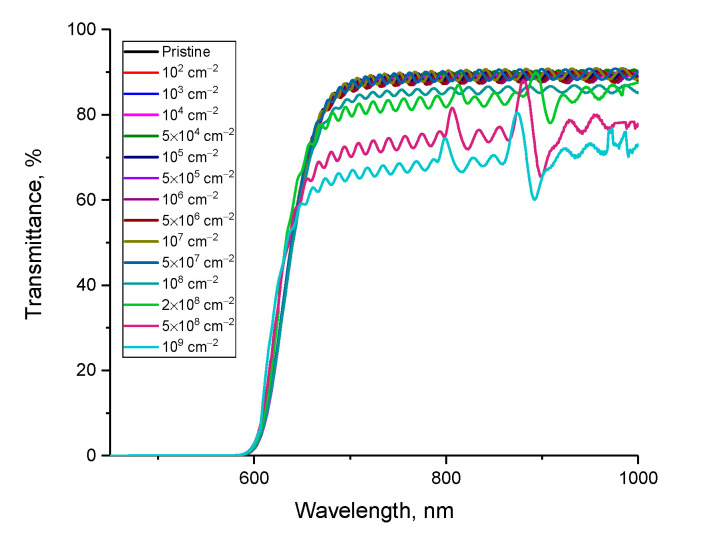
Results of UV spectroscopy of the studied film detectors depending on the density of latent tracks of α-particles.

**Figure 5:**
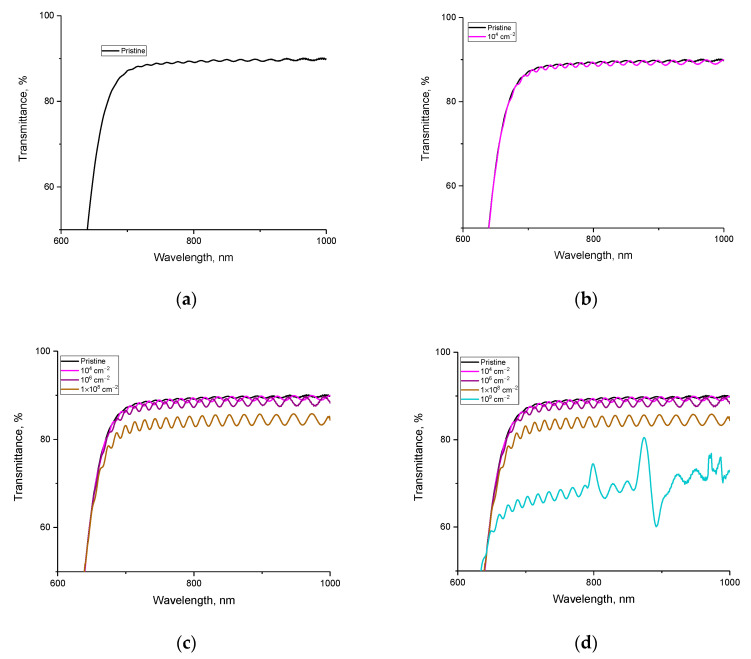
Changes in optical transmission spectra depending on the density of latent tracks: (**a**) initial transmission spectra; (**b**) comparison of the transmission spectra in the initial state and those irradiated with a density of 10^4^ track/cm^2^; (**c**) comparison of the transmission spectra in the initial state and those irradiated with densities of 10^4^, 10^6^, and 10^8^ track/cm^2^; (**d**) comparison of the transmission spectra in the initial state and those irradiated with densities of 10^4^, 10^6^, 10^8^, and 10^9^ track/cm^2.^

Figure 4 shows the results of optical transmission spectra taken in the wavelength range from 450 to 1000 nm, reflecting changes in the optical properties of the studied films depending on the pore density, calculated using surface image analysis. The general view of the transmission spectra indicates the presence of a fundamental absorption edge in the region of 550–570 nm, as well as a high transmission capacity in the region above 650 nm for the films under study. At the same time, as can be seen from the data presented, the changes in the optical spectra depending on the density of latent tracks have a different character, which can be described by several stages of changes in the optical properties. A detailed representation of all observed changes is presented in Figure 5.

In the case of the original sample of film detectors not subjected to irradiation in the transmission region above 650 nm, a small interference of the bands is observed, which, as is known, is characteristic of the molecular structure of the polymer. Changes in the interference pattern subsequent to external influences will be due to a change in the reflection coefficient, refractive index, and optical density of polymer films. The change in these values is primarily associated with the formation of structural changes in the form of deformation distortions along the trajectory of charged particles, as well as the consequences of changes in the electron density and the formation of its anisotropy. At the same time, for latent track densities below 10^4^ track/cm^2^, no changes were observed either in the interference pattern or in the shift of the fundamental absorption edge. From this, we can conclude that the lower limit of sensitivity of optical spectra to structural and electronic changes caused by the interaction of α-particles with a molecular structure is below the density of 10^4^ track/cm^2^.

At a latent track density of 10^4^ track/cm^2^, the optical spectra show a growth in the amplitude of interference fringe oscillations, which indicates that changes occur in the molecular structure of the polymer that can affect the optical properties of the material. The change in the amplitude for irradiated films can be associated with the formation of structural changes associated with distortions of molecular chains in the polymer, as well as the formation of local inhomogeneities in changes in the electron density along the trajectory of charged particles. The occurrence of such an anisotropy in the distribution of electrons upon irradiation with charged particles was reported in [26]. At the same time, an increase in the density of latent tracks leads not only to a growth in the amplitude of oscillations of the interference fringes (see Figure 5c), but also to a decline in the overall transmission intensity at densities above 10^6^ track/cm^2^. This pattern of change can be associated with both an increase in latent track density resulting in an increase in distorted structural regions in the polymer and a decrease in distance between these structurally distorted regions. In this case, according to the data of scanning electron microscopy (see the data in Figure 1), at the densities of latent tracks (taking into account their etching), the distance between the tracks decreases, and there are also areas where latent tracks are quite close to each other (less than 0.1 µm).

In this regard, based on the results of [27] concerning the dependence of the etching rate of latent tracks and their geometry, it can be concluded that the diameters of the structurally changed regions of latent tracks are much larger than those shown in [26]. At the same time, these structurally changed regions have a pronounced anisotropic electron density, the distortion of which is expressed as a change in the transmission value and, as a result, a change in the optical density.

With a growth in the density of latent tracks above 2 × 10^8^ track/cm^2^, a strong decrease in the transmission value is observed, as well as a violation of the amplitude of the oscillations of the interference lines with the formation of a characteristic maximum and minimum in the wavelength range of 870–910 nm. Such changes can be explained by the formation of the effect of maximum convergence of structurally changed regions with each other at high densities of latent tracks, as well as the formation of structural distortions caused by irradiation associated with deformation stresses of molecular chains with a changed electron density.

**Figure 6:**
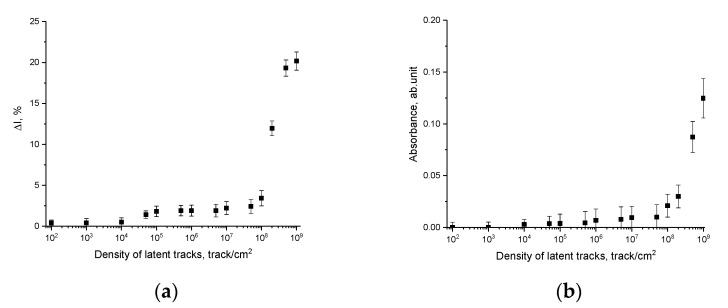
(**a**) Changes in the amplitude of oscillations of interference fringes ∆*I_T_* depending on the density of latent tracks; (**b**) change in the optical density value depending on the density of latent tracks.

Based on the presented changes in the vibration amplitude of the interference fringes, the parameter of the change in the amplitude of the vibrations of the interference fringes ∆*I_T_* was calculated, which is used to numerically determine the amplitude changes subsequent to changes in the density of latent tracks in the polymer. As can be seen from the presented data on changes in the ∆*I_T_* value (see Figure 6a), the most pronounced changes are observed for densities above 10^5^–10^6^ track/cm^2^; however, in the density range of 10^5^–10^8^ track/cm^2^, changes in the ∆*I_T_* value are less than 3%. At the same time, a growth in the density of latent tracks above 10^8^ track/cm^2^ results into a sharp change in the value of ∆*I_T_*, which indicates a change in the optical density and properties of the polymer, which are expressed in the distortion of the molecular chains of the polymer and deformation distortions.

Figure 6b demonstrates changes in the optical density value (absorbance), reflecting the change in the absorption capacity of the material depending on external influences, including irradiation, and also associated with the accumulation of structurally changed areas.

Results of changes in the optical density indicate that a growth in the density of latent tracks has the strongest effect at densities above 10^8^ track/cm^2^, for which changes in the optical density are clearly manifested. At the same time, an increase in optical density correlates well with a change in the amplitude of oscillations of the interference fringes, which indicates that the change in optical characteristics has a clear dependence on the concentration of structurally changed regions and changes in the electron density along the trajectory of latent tracks.

**Figure 7:**
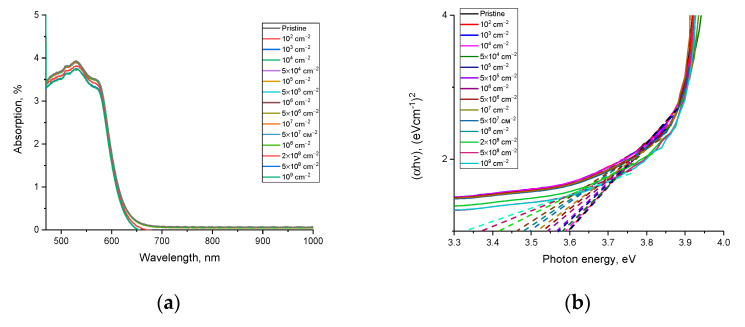
(**a**) Results of optical absorption spectra of spectral lines depending on the density of latent tracks; (**b**) results of changes in the fundamental absorption edge depending on the density of latent tracks.

Figure 7a shows the results of changes in the optical absorption spectra depending on the density of latent tracks in polymer film detectors, which reflect the formation of spectral absorption bands with an increase in the density of latent tracks.

The general view of the optical absorption spectra is characterized by the presence of a wide spectral band in the region of 450–650 nm, with characteristic maxima of the spectral lines at 509 nm (2.43 eV) and 531 nm (2.33 eV), the presence of which can be explained by absorption centers. In this case, a change in the density of latent tracks results into a growth in the intensity of the spectral absorption bands with a slight shift of the maxima, which indicates a change in the concentration of absorbing centers. Based on the obtained optical absorption spectra using Tauc plots, the values of the band gap were calculated. The results of the constructions are shown in Figure 7b.

**Figure 8:**
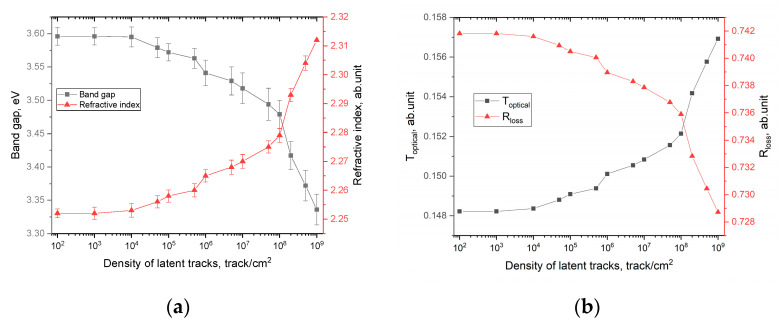
(**a**) Results of changes in the band gap and refractive index; (**b**) data on changes in *T_optical_* and *R_loss_* values depending on the density of latent tracks.

The overall appearance of the presented constructions indicates that with an increase in the density of latent tracks, the fundamental absorption edge shifts to the region of lower energies, which indicates a change in the electron density, as well as the formation of additional absorption centers. Based on the tangents drawn, the values of the band gap and the refractive index were calculated. The calculation results are shown in Figure 8a.

The general nature of the change in the values of the band gap and the refractive index, which have mutual feedback with each other, shows the dependence of changes on the density of latent tracks, as well as changes associated with them. At the same time, the most pronounced changes in these values, in contrast to the calculated values of optical density and changes in the amplitude of interference fringes, are observed for densities of 10^4^ track/cm^2^, while a change in optical density values was observed at higher densities of latent tracks. This difference in changes in optical characteristics can be explained by the effects caused by irradiation and the consequences they create. Due to the dielectric nature of polymer films, the change in the electron density near the trajectory of latent tracks is not reversible, or the time of reversible relaxation after irradiation (i.e., restoration of the electron density and electron distribution) is very high. According to [26], such changes persist for several years without visible changes. At the same time, taking into account the data on the dependence of the etching rate of latent tracks taken from their work [28], it can be concluded that structurally changed regions or regions with a changed electron density are much larger. At the same time, these changes themselves can be anisotropic in nature along the depth of the perpendicular trajectory of charged particles in the material. This nature of latent tracks with a changed electron density affects the change in the band gap and, as a consequence, in the refractive index, a growth in which indicates an increase in the concentration of absorbing centers in the irradiated polymer, which is more pronounced at high densities of latent tracks. Additionally, in the case when these areas are close to overlapping, the changes in the values of the optical characteristics become the most pronounced, both in the case of a change in the optical density and an increase in the refractive index, and a decrease in the band gap.

Based on changes in the band gap and refractive index, optical characteristics such as optical transmission and reflection loss were calculated. The calculation results are shown in Figure 8b. From the presented results of estimating these values, it can be concluded that a growth in the density of latent tracks, leading to the formation of local structurally changed regions, results into a slight change in the optical losses of the transmission and reflection values. However, when the distance between structurally changed regions becomes smaller, the contribution of reflection losses increases, and as a result, a decrease in the optical transmission of film detectors is observed.

Summarizing the general changes in the optical characteristics of film detectors depending on the density of latent tracks, we can conclude that at low densities (below 10^4^ track/cm^2^), the use of optical spectroscopy to assess changes caused by irradiation is not appropriate. This can be explained by the fact that at given densities of latent tracks, the local structurally changed regions caused by them, which appear along the trajectory of charged particles, are located at a sufficiently large distance from each other. This does not allow one to fix small changes in the electron density and optical characteristics caused by this interaction. In this case, based on the data of scanning electron microscopy of the surface of film detectors subjected to chemical etching, it can be concluded that the distances between local structurally changed regions are on the order of several tens of microns. However, with an increase in the irradiation density above 10^4^ track/cm^2^, changes are observed both in the interference lines of the optical spectra and in changes in the optical characteristics. At the same time, these changes in the optical values at irradiation densities above 10^7^ track/cm^2^ allow us to conclude that the change in the optical properties manifests itself not only in a change in the optical characteristics, but also in a decline in the transmission value, which indicates a significant change in the optical density and electron density distribution. In this regard, it can be assumed that the sizes of structurally changed regions that appear along the trajectory of charged particles are much larger than previously reported in [26,29].

Another way to evaluate the changes in materials caused by irradiation of charged particles is the X-ray diffraction method, which, as in the case of the optical spectroscopy method, belongs to non-destructive methods for monitoring structural changes. This method is based on the description of structural changes in materials associated with distortions and deformations of the crystal structure subsequent to the accumulation of point defects, areas of disorder, etc. At the same time, the use of this method to study structural changes in polymer films subsequent to irradiation showed good agreement between the observed formations of structurally changed regions and deformation distortions of molecular chains in polymer films with optical spectroscopy data [24,25,26]. In this regard, using the data on changes in the structural parameters determined using the X-ray diffraction method, it is possible to describe the observed changes in the optical properties of film detectors exposed to irradiation of α-particles.

**Figure 9:**
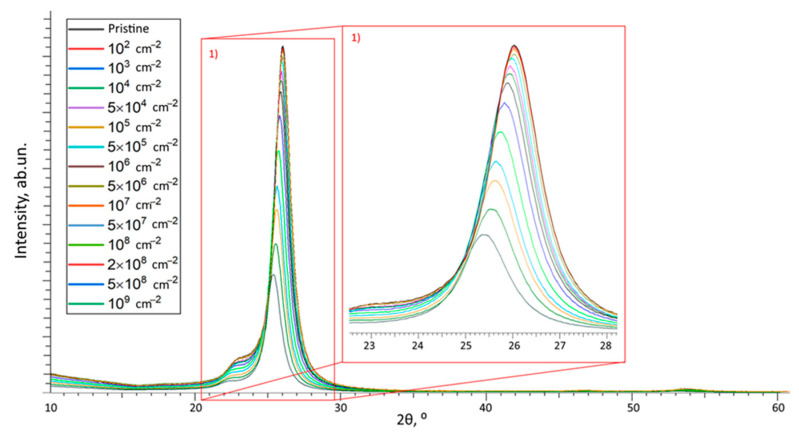
Results of X-ray diffraction of the studied film samples depending on the irradiation density.

Figure 9 shows X-ray diffraction patterns that reflect changes in the intensity and position of the main diffraction peak with a maximum of 2θ = 25.8°, which characterizes the crystalline component of the polymer film used as a nuclear-tracking detector. X-ray diffraction patterns were taken in the Bragg–Brentano geometry, in the angular range 2θ= 10–60°, reflecting changes in the polymer film and characterizing its crystalline and amorphous components of the polymer.

The general trend of changes in diffraction reflections depending on the density of latent tracks is characterized by two types of changes: (1) a decline in the intensity of the main reflection, and as a result, a change in the half-width of the diffraction line (FWHM); (2) shift of the diffraction maximum to the region of small angles, indicating a change in the deformation distortions of molecular chains subsequent to the interaction of α-particles with the polymer structure.

At the same time, as in the case of changes in optical characteristics, when analyzing changes in the intensities of diffraction reflections, their shifts, and FWHM, it was found that the most pronounced changes are observed for samples with latent track densities above 10^4^ track/cm^2^. This observation indicates that the use of the X-ray diffraction method has a sensitivity threshold in the difference between structural changes caused by the interaction of α-particles, which is at least 10^4^ track/cm^2^.

The assessment of structural changes associated with the formation of latent tracks, as well as a change in the electron density. Structural changes caused by irradiation are due to a change in the configuration of molecular chains, which occurs when the electron density changes subsequent to the formation of latent tracks in the material. In the case of the interaction of incident charged particles with a material, an anisotropy of electron density is formed along the trajectory of the particle with the formation of the so-called δ-electrons, which initiate ionization processes and electronic excitations along the trajectory of passage of a charged particle at a sufficiently large distance from the center [30]. The formation of δ-electrons and their subsequent migration results into an anisotropic distribution of electron density, which in a dielectric leads to deformation distortions of molecular chains, which is expressed in the form of structural changes.

Such structural changes can be assessed by comparative analysis of the shape of diffraction reflections, as well as its intensity, which serves as a good indicator of deformation. This rating is based on the following factors. Firstly, structural parameters are extracted from the nature of the interaction of X-rays with matter. Any deviation from the reference values, or in the case of serial measurements associated with the assessment of external influences on the structure of the material from the original sample, is associated with deformation of the structure caused by external influences or other factors. In this regard, direct visualization of the comparison of diffraction patterns of samples with different types of exposure can allow us to evaluate the effect of this exposure [31,32]. Secondly, the deviation of the shape of diffraction reflections from symmetry relative to the maximum, while maintaining the FWHM value, indicates a deformation distortion of the structure, which is expressed in the creation of additional obstacles in the diffraction of X-rays. Additionally, a change in the FWHM value, considering changes in the intensity of diffraction reflections, in some cases makes it possible to estimate the degree of amorphization of the material associated with a change in the molecular structure due to the breaking of chemical bonds and the appearance of free radicals [33,34]. Thirdly, analyzing the shift of the maximum of the diffraction reflection relative to the initial position indicates the nature and type of deformation distortions that occur in the irradiated material (tensile or compressive stresses). Determination of the type of deformation distortions, as well as the dynamics of their change depending on the magnitude of external influences, allows evaluation of dynamics of the accumulation of structural changes, and the subsequent reaction of the material to these influences.

Thus, by analyzing the obtained X-ray diffraction patterns, one can find out the nature of the structural changes caused by irradiation without destroying the test sample or any manipulations with the samples that lead to its destruction.

**Figure 10:**
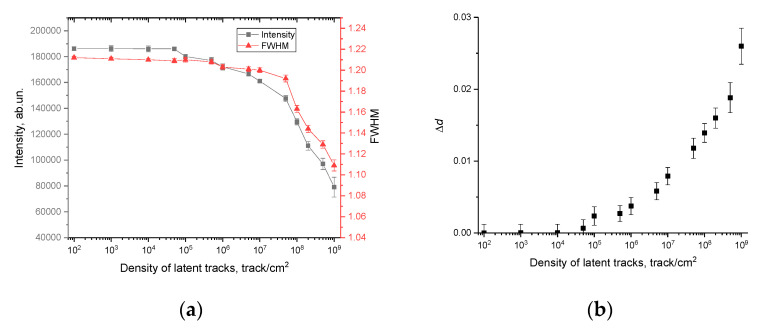
(**a**) Estimation results of the change in the intensity of the diffraction reflection at 2θ = 25.8° and its FWHM depending on the density of latent tracks; (**b**) estimation results of the change in the value of Δ*d* depending on the density of latent tracks.

Figure 10a demonstrates the assessment results of the change in the diffraction reflection intensity, as well as the FWHM values, which reflect the formation of structurally changed inclusions in the structure of the polymer subjected to irradiation.

The general view of the presented dependence of the change in intensity and FWHM indicates a direct dependence of structural distortions on the density of latent tracks of registered α-particles at different exposure times. At the same time, as in the case of a change in the optical characteristics of irradiated films, for changes in the main characteristics of X-ray diffraction patterns, there is a sensitivity threshold of 10^4^ track/cm^2^, below which no structural changes, expressed in a change in the intensity of the diffraction reflection and FWHM, have been established. The presence of a sensitivity threshold for X-ray diffraction, as in the case of optical methods, is due to the following fact. During interaction of incident charged particles with the molecular structure of the polymer, the main contribution to structural changes is made by changes in the distribution of electron and charge density in the material, which in the case of polymer molecular chains can be expressed in a change in their configuration. In the case of low densities, these changes are isolated from each other at a sufficiently large distance and are also few in number due to the low density of latent tracks, which does not allow them to be distinguished from the overall picture of structural and optical characteristics. With an increase in the density of latent tracks (above 10^4^ track/cm^2^), when both the number of structurally changed regions becomes larger and the average distance between these regions is smaller, such changes can be recorded both by optical and X-ray methods of analysis.

As can be seen from the data presented, alterations in the intensity and FWHM value depending on the density of latent tracks are of a different nature. A significant change in the FWHM value is not observed up to a density of 10^7^–5 × 10^7^ track/cm^2^, while a change in the intensity of the diffraction reflection at 2θ = 25.8° is already observed for a density above 10^4^ track/cm^2^. At latent track densities above 5×10^7^ track/cm^2^, the change in intensity and FWHM values have the same downward trend. Such a difference in changes in the range of latent track densities from 10^4^ to 10^7^ track/cm^2^ is due to the difference in the mechanisms of structural changes caused by the accumulation of latent tracks and the consequences of their interaction with the molecular structure of the polymer. At low densities of latent tracks, the main changes in the intensity, in the position of the maximum of the diffraction reflection, are associated with deformation distortions caused by changes in the electron and charge density in latent tracks during the interaction of charged particles with the polymer. At the same time, the isolation of these structurally changed regions causes deformation distortion, without a visible change in the ratio of the crystalline and amorphous (structurally disordered) part of the irradiated polymer. At latent track densities above 10^7^ track/cm^2^, when the average distance between the structurally changed areas of latent tracks becomes small, the structurally changed areas approach each other, which results into more significant changes in the molecular structure of the polymer. In the case of structural changes, this may be due to strong deformation distortions of molecular chains, the appearance of an anisotropic distortion in the electron density, as well as effects associated with the destruction of chemical bonds.

Figure 10b shows the alteration results of the diffraction maximum position shift Δ*d* depending on the density of latent tracks, the change in which characterizes the deformation distortions that occur in the polymer structure after irradiation.

## 4. Discussion

The general view of the presented structural changes indicates that the most pronounced changes associated with deformation distortions appear at latent track densities above 10^4^ track/cm^2^, and the dynamics of changes itself has a pronounced dependence on the density of latent tracks. At the same time, the nature of the change in the value of Δ*d* depending on the density of latent tracks indicates that deformation distortions are characteristic of tensile strains, expressed in a growth in Δ*d*.

**Figure 11:**
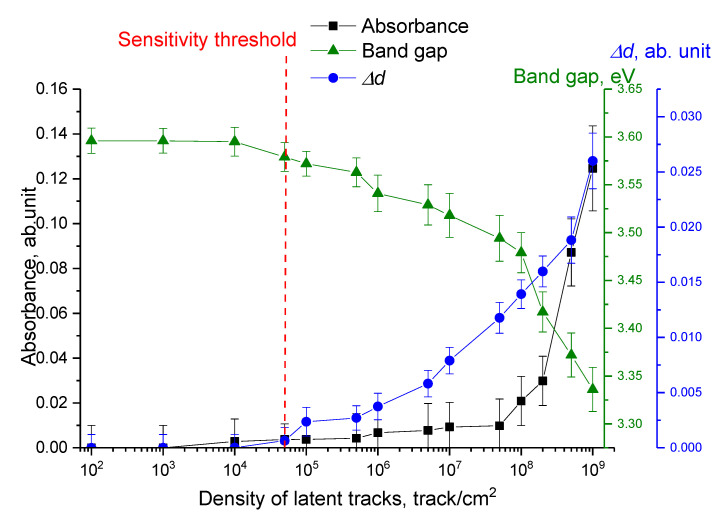
Results of a comparative analysis of changes in optical and structural parameters determined for film detectors with different latent track densities.

To assess the applicability of the methods of optical spectroscopy and X-ray diffraction, Figure 11 shows the comparison results of the optical density, band gap, and Δ*d* values, which reflects the deformation distortion of the structure.

As is evident from the data presented, alterations in the optical and structural parameters correlate quite well with each other, and the structural changes in the Δ*d* value and the band gap width have a common trend of changes. Moreover, by comparing the structural and optical changes, it is possible to determine the sensitivity threshold of these analysis methods, which amounted to 10^4^ track/cm^2^, i.e., at latent track densities below this threshold, no change in the optical and structural characteristics of the polymer is observed. If the density of latent tracks exceeds this value, the changes in the optical and structural characteristics are clearly distinguishable, and by their changes it is possible, using the dependences in Figure 11, to determine the density of latent tracks, and to establish the concentration dependences of radon and its daughter decay products.

The applicability of optical spectroscopy and X-ray diffraction as methods for assessing changes in the optical and structural properties of polymer films caused by exposure to ionizing radiation has been actively studied in the past few years. Moreover, interest in this topic is due not only to the search for fundamental aspects of the relationship between structural changes caused by the interaction of charged particles with the molecular structure of polymers, but also to the search for new aspects of the practical application of irradiated films. For example, in a series of works [23,24,25,35,36,37], a detailed description is given of the connection between structural changes caused by irradiation with heavy ions in polymer films and the optical properties of polymers, including changes in the fundamental absorption edge, electron density, transmission, etc. At the same time, the authors of these works demonstrated that the variation of irradiation conditions results into the formation of anisotropic changes in the electron density in latent tracks, which is in good agreement with the data of [26]. The paper [36] also shows the prospects for using irradiated polymer films as electret materials, and the authors attribute the appearance of electret properties in films to its redistribution and accumulation during polarization of PET films after irradiation with accelerated fast ions. At the same time, the emphasis in the study of latent tracks and related changes is not on the visualization of tracks, but on the study of their properties and the relationship between optical, electronic, and structural changes. Much attention is paid to determining the nature of these changes and their interpretation. In this case, this work shows the lower limit of applicability of the methods of optical spectroscopy, as well as X-ray diffraction in the detection of latent tracks in polymer nuclear track detectors, which can later be used as a calibration curve in the case of full-scale tests in the detection and determination of the concentration dependences of radon.

## 5. Conclusions

The paper presents evaluation results of the applicability of optical spectroscopy and X-ray diffraction to determine the density of latent tracks of α-particles emitted by the Am-241 source. During the studies, the dependences of changes in optical characteristics, such as optical density, band gap (fundamental absorption edge), and refractive index depending on the density of latent tracks and the structural consequences caused by them in the molecular structure of the polymer, were established. The dependences of changes in structural characteristics associated with deformation distortions of molecular chains and, therefore, changes in the electron and charge density in the material depending on the density of latent tracks are determined. The sensitivity thresholds for determining the density of latent tracks were determined using X-ray diffraction and optical spectroscopy methods.

The proposed methods for registering the total activity of alpha-emitting radionuclides, as well as determining the absorbed dose, will increase the accuracy of determining these values for long exposure times, as well as determine the dynamics of changes in the background radiation in selected areas of study.

In the future, the results obtained will be used in the form of calibration curves to determine the concentration dependences of radon from the density of latent tracks obtained in field experiments.

## Data Availability

Not applicable.
